# The role of cochlear implantation in alleviating Tumarkin drop attacks of Meniere's disease; a case report

**DOI:** 10.1016/j.joto.2023.06.002

**Published:** 2023-06-26

**Authors:** Chiara Filippi, Edoardo Covelli, Haitham H. Elfarargy, Simonetta Monini, Maurizio Barbara

**Affiliations:** a Department of Neuroscience, Mental Health, and Sense Organs (NEMOS), Sant’ Andrea University Hospital, Sapienza University, Rome, Italy; b Otorhinolaryngology Department, Kafrelsheikh University, Kafrelsheikh, Egypt

**Keywords:** Ménière's disease, Tumarkin drop attacks, Hearing loss, Cochlear implant

## Abstract

Ménière's disease (MD) patients may suffer episodes of sudden falls, named Tumarkin drop attacks (DAs). This fall occurs abruptly and without warning or loss of consciousness. DAs usually aggravate the clinical picture of MD and are challenging to manage. The present report describes a case treated by cochlear implantation (CI) due to concomitant deafness and offers some clinical considerations for this condition. A male patient aged 48 years with a 10-year history of definite bilateral MD had profound SNHL on the right and severe SNHL on the left side. He suffered from intermittent attacks of vertigo, ear fullness, and tinnitus and, in the last year, had developed DAs and experienced 14 episodes in the previous six months. The preoperative category of acoustic performance was 3. The Dizziness Handicap Inventory (DHI) questionnaire showed a total score of 46, which indicated a moderate degree of disability. A CI was planned for the right side. The patient did not report any further DAs episode for two years since then. The postoperative category of acoustic performance became 11, and the postoperative DHI questionnaire showed a decrease in the total score (from 46 to 19), which indicated a mild disability. Unilateral CI effectively alleviated the DAs associated with bilateral MD. Our report proposes a new modality for managing vertiginous symptoms in cases of MD with hearing loss without the need for more aggressive surgical interventions with the need for clinical trials to confirm our results.

## 1 Introduction

Ménière's disease (MD) is a challenging inner ear pathology. It is clinically known as a cochleovestibular dysfunction that presents an association of periodic vertigo, ear fullness, tinnitus, and progressive sensorineural hearing loss (SNHL) (Berlinger, 2011). Deafness tends to be low-frequency and fluctuating at the start of the pathology, with eventual stabilization as a moderate-to-severe grade. In addition, a consistent partition of this population may become profound deafness with communication disabilities, especially when a bilateral involvement is shown. The severity of symptoms varies between patients and even for the same patient over time. In some patients, vestibular symptoms can be severe, with less effect on hearing or vice versa (Selleck et al., 2021).

Ten percent of MD patients suffer from episodes of sudden falls known as drop attacks (DAs) (Black et al., 1982). DAs associated with MD were called an “otolithic catastrophe” by Tumarkin in 1936. These DAs occur suddenly and without loss of consciousness at any time in daily life, with the potential risk of injury (Baloh et al., 1990; Janzen and Russell, 1988). Various treatment modalities have been proposed for managing MD, such as conservative treatment, labyrinthectomy, endolymphatic shunt operation, vestibular neurectomy, intratympanic steroids, and intratympanic gentamicin (ITG) administration. MD's treatment options concentrate on lowering vestibular symptoms while maintaining hearing; the most effective management plan is decided case by case as each modality has some risks, which may impact the outcomes and the quality of life (Perez-Fernandez et al., 2010; Viana et al., 2014).

Cochlear implantation (CI) has become a standard option for managing SNHL associated with MD. CI can increase receptive communication without adversely impacting vestibular function. However, it could be more challenging if hearing loss is associated with severe vestibular manifestations such as vertigo, DAs, or both. In this case, CI may accompany simultaneous labyrinthectomy or vestibular neurectomy (Desiato et al., 2020).

This case reports on the outcome of a cochlear implant in a patient with asymmetric SNHL with regard to hearing and control of vestibular symptoms and drop attacks.

## 2 Case report

### 2.1 History

A male patient aged 48 years presented with a 10-years history of definite bilateral MD. He suffered intermittent attacks of rotatory vertigo, ear fullness, and tinnitus and showed bilateral hearing loss, which deteriorated progressively over three years. In the last year, he developed DAs, with 14 episodes in the previous six months. The attacks occurred suddenly without warning, and, during all the attacks, he remained conscious without vomiting or nausea. Each attack lasted less than one minute, followed by a full recovery. These episodes did not respond to a conservative medical treatment based on a low-salt diet and diuretics. No systemic diseases were reported, but the patient, due to vestibular manifestations, had to stop driving. All neurological examinations were within normal. These attacks resulted in stopping driving before CI surgery.

### 2.2 Preoperative investigations

**Fig. 1:**
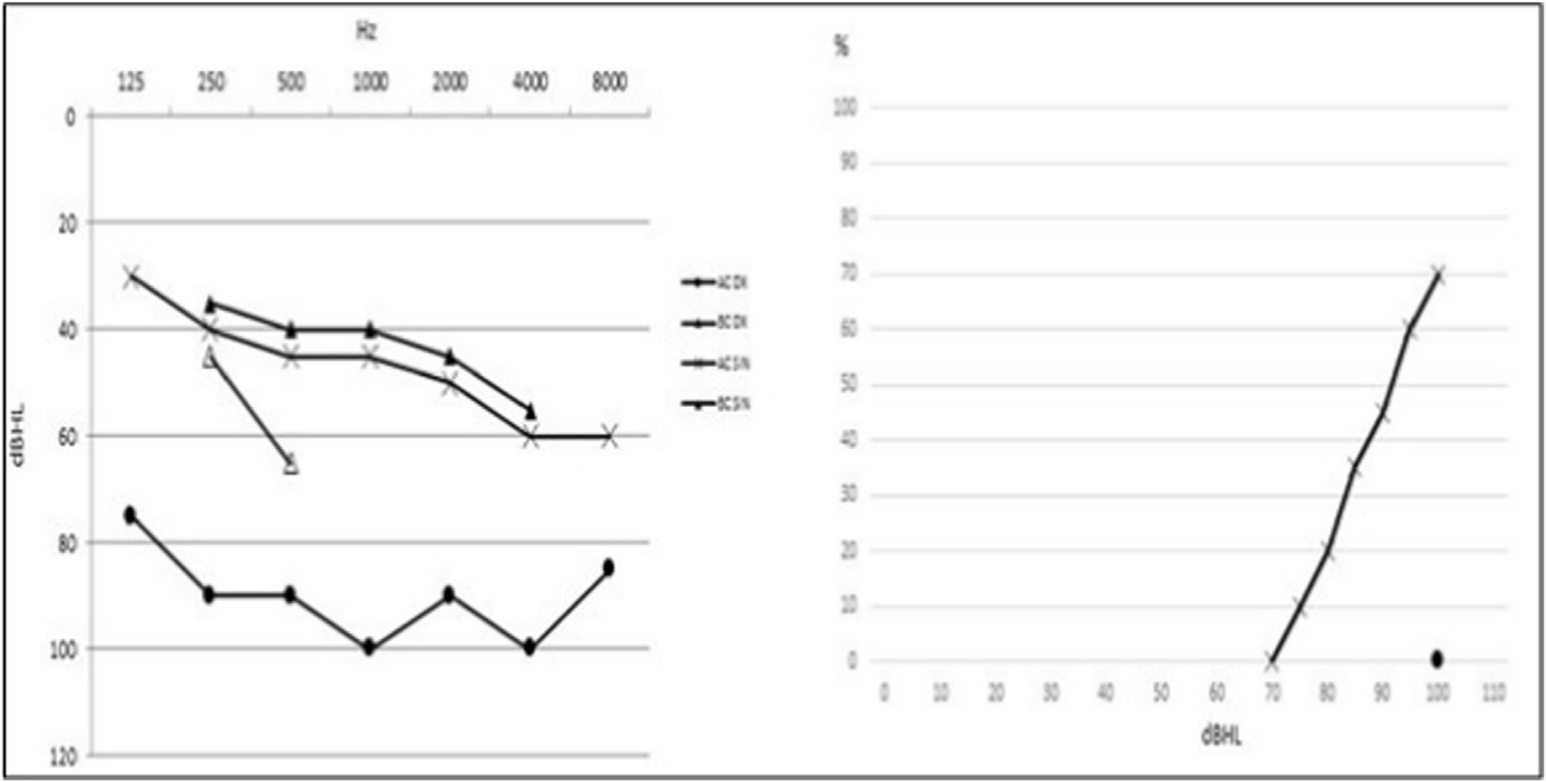
Pure tone audiometry and speech audiometry in headphones before CI. (Ac: air-conduction, Bc: bone-conduction, DI: right ear, SIN: left ear).

Baseline pure tone audiometry and speech audiometry showed an asymmetric sensorineural hearing loss of severe degree on the left ear and profound on the right one. The speech recognition was 70% at 100 dB HL on the left side and 0% at 100 dB HL on the right side. The sound field's unaided pure-tone average (PTA) (250–4000 Hz) threshold was 75 dB HL, and speech recognition was 30% at 75 dB HL (Fig. 1). The patient wore a behind-the-ear hearing aid on the left ear for five years, with scarce benefit. The preoperative category of auditory performance was 3 (Guo et al., 2014).

The vestibular investigations included the video head impulse test (v-HIT) (ICS Impulse, Otometrics, Taastrup, Denmark) and the air-conduction sound (tone burst) stimulated cervical vestibular-evoked myogenic potentials (C-VEMPS) (Eclipse, Interacoustic, Eden Prairie, USA). These vestibular investigations did not display impairment on either side. By v-HIT, the presence of overt or covert saccades was searched (Table 1) (Fig. 3). For C-VEMPs, the latency of p13 and n23 peaks were identified. The Dizziness Handicap Inventory (DHI) questionnaire showed a total score of 46, which indicated a moderate degree of disability (Jacobson and Newman, 1990).

**Table 1:** The air-conduction C-VEMPs in the preoperative evaluation, fifteen days after the operation, and one year after. (C-VEMPs: cervical vestibular-evoked myogenic potentials, P1: positive wave, N1 negative wave, msec: milli-second).

	Preoperative C-VEMPs		C-VEMPs 15 after surgery		C-VEMPs one year after surgery	
	RIGHT	LEFT	RIGHT	LEFT	RIGHT	LEFT
**P1 (msec)**	14	13	14	15	13	14
**N1 (msec)**	22	23	23	23	22	22

**Fig. 2:**
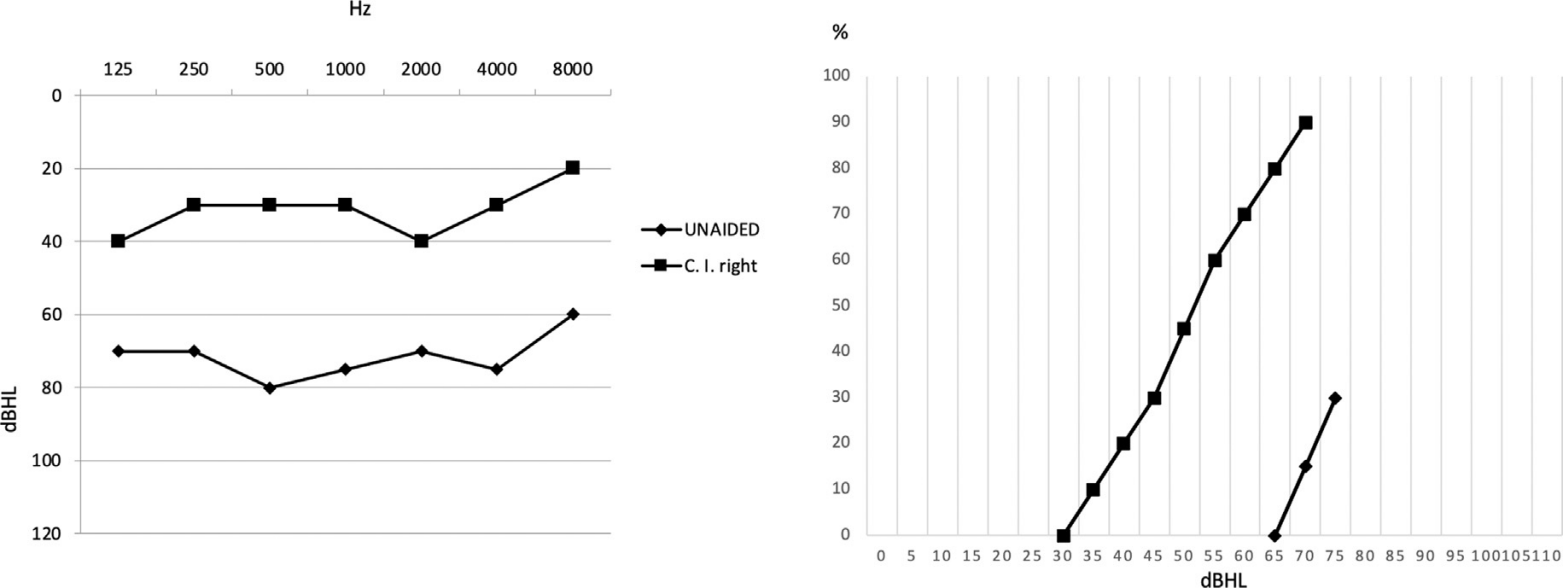
Pure tone audiometry and speech audiometry in free field, unaided and with CI turned on, one year after CI.

**Fig. 3:**
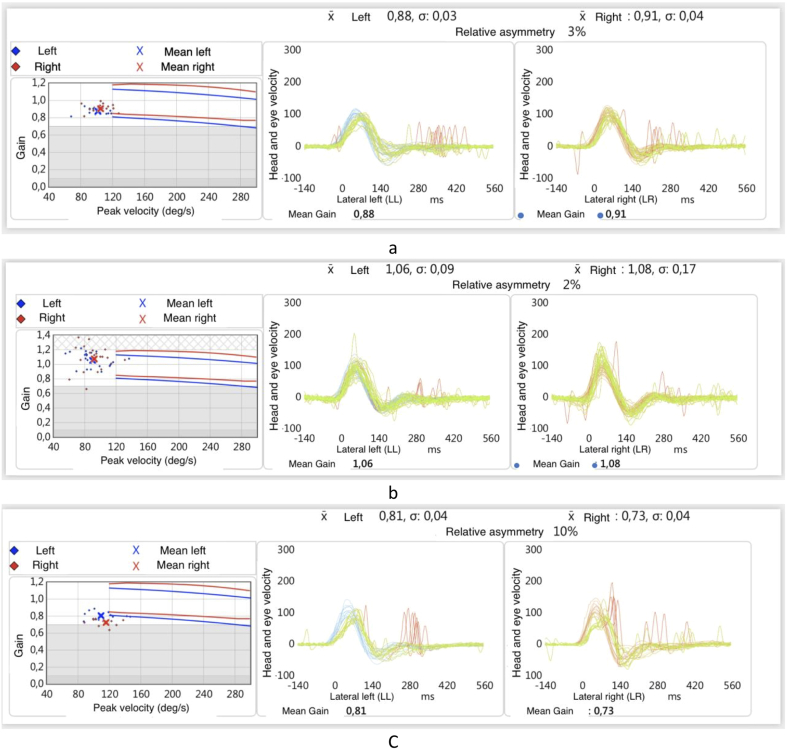
Vestibular-ocular reflex (VOR) gain at video-head impulse test (v-HIT) before surgery, 15 days after surgery (CI off), and one year after surgery (CI on). (LSC: Lateral semicircular canal, LARP: left anterior right posterior, RALP: right anterior left posterior).

Magnetic resonance imaging (MRI) of both ears showed normal morphology of the cochlea and normality at the cerebellopontine angle level.

Due to the hearing situation, the patient was arranged to undergo CI on the worse side (right ear). Therefore, the CI was performed with a Synchrony FLex28 (Med-el, Innsbruck, Austria) via the round window membrane approach, with no intra- or postoperative complications, after getting the signed informed consent.

### 2.3 Postoperative outcome

The implant was activated forty days after the operation. The audiometric findings 1-year after surgery showed a sound field PTA (250–4000 Hz) gain of 43 dB HL with improved speech recognition by 60% without fluctuations (Fig. 2). The postoperative category of acoustic performance was 11.

The v-HIT and C-VEMPS were performed 15 days after surgery when the implant was still inactive and one year after. Both revealed no significant changes. The patient did not report any further DAs episodes in the two years following CI. The postoperative DHI questionnaire showed a decrease in the total score, as it was 19, which indicated a mild disability. The patient could drive again and return to social activities without significant disequilibrium problems.

## 3 Discussion

In the last two decades, CI has been increasingly used in managing SNHL associated with MD. The audiological results are acceptable and comparable to the CI patients without MD. There is improving in speech discrimination with minimal fluctuations. The vestibular manifestations associated with MD can be managed by CI only, CI with simultaneous labyrinthectomy, or labyrinthectomy with sequential CI in cases with severe to profound hearing loss. The direct impact of CI on vestibular symptoms needs to be clarified. Possible explanations for improving vestibular symptoms after CI may include a change in the inner ear fluid homeostasis or an inflammatory state with consequent fibrosis due to the electrode itself, which might alter the endolymphatic flow in the semicircular canal. Another possibility is the intraoperative perilymph loss due to opening the round window, leading to a reduction in scala tympani pressure and, therefore, causing the basilar membrane to be displaced downwards; Frodlund et al. showed how the round window and the use of straight electrodes could cause intracochlear trauma associated with the development of fibrosis of the ductus reuniens and, therefore, with alterations of endolymphatic homeostasis (Villavisanis et al., 2021; Liu et al., 2016; Wu et al., 2019; Shi and Kertesz, 2015; Heywood and Atlas, 2016; Huang and Young, 2012; Barbara et al., 2020; Frodlund et al., 2016). Although there is a decrease in frequency, outcomes for dizziness and vertiginous presentations differed obviously between various studies and should be treated on a case-to-case trial (Desiato et al., 2020).

One of the reported vestibular manifestations associated with MD is DAs, whose pathophysiological mechanism is unknown. The description of a sudden push or shove and an illusion of linear displacement or tilt of the environment indicates a premature stimulation of the otolithic organs. This could result from a mechanical defect due to pressure differences within the inner ear or a rapid alteration in the electrolyte composition of the endolymph due to the injury of the membranous labyrinth. The rapid transmission of neural waves from the otolith would be directed into the vestibulospinal reflex pathways and cortical areas that control spatial orientation. A sudden fall could be caused directly by vestibulospinal stimulation of motor neurons or indirectly from motor reflexes motivated by higher centers (Baloh et al., 1990).

DAs occurring as sudden falls without warning are critical and require adequate treatment. Liu et al. used intratympanic dexamethasone injection as the main treatment for MD patients with DAs. They found that DAs in 71.4% of patients improved after primary management (Liu et al., 2016). Qianru et al. used intratympanic gentamicin injection for controlling DAs in 13 MD cases. He achieved control at 92.31% (Wu et al., 2019). In both previous studies, MD was unilateral without significantly impacting hearing. There needed to be a long-term follow-up of this progressive disease. Additionally, although gentamicin is mainly a vestibulotoxic drug, there is still a high risk to the inner ear, with a recording of hearing worsening in 5–25% of cases (Shi and Kertesz, 2015).

On the other side, many surgical solutions were tried to control DAs associated with MD. Black FO et al. and Janzen VD agreed that endolymphatic shunt operations effectively manage the DAs. The vestibular nerve section was preferable in cases with hearing, whereas a labyrinthectomy might be put in mind in patients with profound HL (Black et al., 1982; Janzen and Russell, 1988).

There are few studies about the impact of CI on the occurrence and frequency of DAs. In most of these trials, the CI was accompanied by a simultaneous or sequential vestibular neurectomy or labyrinthectomy. This may result in bilateral vestibular dysfunction with subsequent bobbing oscillopsia and the inability to move in dark places (Shi and Kertesz, 2015; Heywood and Atlas, 2016). Shi J et al. reported a case with frequent DAs who suffered from a severe form of MD in the only hearing ear. This patient underwent a CI in the contralateral ear and a sequential vestibular neurectomy of the affected ear. The patient developed a postoperative. oscillopsia (Shi and Kertesz, 2015).

Our study concerned a case with bilateral MD with disabling vertigo and bilateral asymmetrical SNHL. The patient had frequent DAs, which occurred in the late stage of MD, nine years after the incidence of MD. Janzen et al. observed that the period between the onset of MD and the first presentation of a DA ranged from 3 to 20 years (Janzen and Russell, 1988). We tried to avoid the drawbacks of the other modalities. CI effectively prevented DAs without other surgical procedures and preserved the vestibular system without further oscillopsia. The change in the DHI results from moderate to mild with returning to driving and other social activities indicated the efficacy of CI in managing MD-associated vertigo. This effect was beside the main successful audiological impact of CI. The patient became CAP eleven without fluctuations in the next two years. Also, expected gains of VOR were detected by the objective v-HIT.

The presence of C-VEMPs in the preoperative evaluation coincided with what was reported by Huang et al. This may indicate that the action of the otolithic organ in MD cases with DAs may not be dysfunctional entirely and that it is responsive to some unanticipated stimulating signals, such as a sudden alteration in endolymphatic pressure (Huang and Young, 2012).

## 4 Conclusions

According to this case report, CI effectively alleviated the DAs associated with bilateral MD. In addition, it prevented postoperative DAs and improved the patient's vertiginous situation without the other surgical interventions. Moreover, CI effectively managed hearing loss related to bilateral MD with a better postoperative audiological performance without significant fluctuations. However, more clinical trials are needed to confirm our results and evaluate CI's impact in cases with unilateral MD.

## Funding

The authors have no budget or financial relationships to disclose.

## Declaration of competing interest

There is no conflict of interest.

## References

[bib1] Baloh R.W., Jacobson K., Winder T. (1990). Drop attacks with Menière's syndrome. Ann. Neurol..

[bib2] Barbara M., Talamonti R., Benincasa A.T., Tarentini S., Filippi C., Covelli E., Monini S. (2020). Early assessment of vestibular function after unilateral cochlear implant surgery. Audiol. Neurootol..

[bib3] Berlinger N.T. (2011). Meniere's disease: new concepts, new treatments. Minn. Med..

[bib4] Black F.O., Effron M.Z., Burns D.S. (1982). Diagnosis and management of drop attacks of vestibular origin: Tumarkin's otolithic crisis. Otolaryngol. Head Neck Surg..

[bib5] Desiato V.M., Patel J.J., Nguyen S.A., Meyer T.A., Lambert P.R. (2020). Cochlear implantation in patients with Meniere's disease: a systematic review. World J Otorhinolaryngol Head Neck Surg.

[bib6] Frodlund J., Harder H., Mäki-Torkko E., Ledin T. (2016). Vestibular function after cochlear implantation: a comparison of three Types of electrodes. Otol. Neurotol..

[bib7] Guo S., Li H., Chen B., Dai C. (2014). [Study of categories of auditory performance and speech intelligibility rating of post-lingual cochlear implantes]. Lin Chung Er Bi Yan Hou Tou Jing Wai Ke Za Zhi.

[bib8] Heywood R.L., Atlas M.D. (2016). Simultaneous cochlear implantation and labyrinthectomy for advanced Ménière's disease. J. Laryngol. Otol..

[bib9] Huang C.H., Young Y.H. (2012). Ocular and cervical vestibular-evoked myogenic potentials in Tumarkin falls. Otol. Neurotol..

[bib10] Jacobson G.P., Newman C.W. (1990). The development of the dizziness Handicap inventory. Arch. Otolaryngol. Head Neck Surg..

[bib11] Janzen V.D., Russell R.D. (1988). Conservative management of Tumarkin's otolithic crisis. J. Otolaryngol..

[bib12] Liu B., Leng Y., Zhou R., Liu J., Liu D., Zhang S.L., Kong W.J. (2016). Intratympanic steroids injection effectively treats drop attacks with Ménière's disease and delayed endolymphatic hydrops: a retrospective study. Medicine (Baltim.).

[bib13] Perez-Fernandez N., Montes-Jovellar L., Cervera-Paz J., Domenech-Vadillo E. (2010). Auditory and vestibular assessment of patients with Ménière's disease who suffer Tumarkin attacks. Audiol. Neurootol..

[bib14] Selleck A.M., Dillon M., Perkins E., Brown K.D. (2021). Cochlear implantation in the setting of Menière's disease after labyrinthectomy: a Meta-analysis. Otol. Neurotol..

[bib15] Shi J., Kertesz T. (2015). Contralateral cochlear implantation prior to vestibular nerve section for 'drop attacks' in the only hearing ear. J. Laryngol. Otol..

[bib16] Viana L.M., Bahmad F., Rauch S.D. (2014). Intratympanic gentamicin as a treatment for drop attacks in patients with Meniere's disease. Laryngoscope.

[bib17] Villavisanis D.F., Mavrommatis M.A., Berson E.R., Bellaire C.P., Rutland J.W., Fan C.J., Wanna G.B., Cosetti M.K. (2021). Cochlear implantation in Meniere's disease: a systematic review and Meta-analysis. Laryngoscope.

[bib18] Wu Q., Li X., Sha Y., Dai C. (2019). Clinical features and management of Meniere's disease patients with drop attacks. Eur. Arch. Oto-Rhino-Laryngol..

